# The Signal-Integrity Control Strategy of a TSV Array for a Chiplet-Based System

**DOI:** 10.3390/mi17070822

**Published:** 2026-07-10

**Authors:** Bosen Wang, Hongjian Su, Shengqi Zhang, Di Li, Dongdong Chen, Yintang Yang

**Affiliations:** 1 Faculty of Integrated Circuit, Xidian University, Xi’an 710071, China; bisonwang@stu.xidian.edu.cn (B.W.); 25251215854@stu.xidian.edu.cn (H.S.); dili@xidian.edu.cn (D.L.); 2 The Shaanxi Key Laboratory of Integrated Circuits and System, Xi’an 710071, China; 3 School of Applied Science, Beijing Information Science & Technology University, Beijing 102206, China; 2024021183@bistu.edu.cn

**Keywords:** Chiplet-based system, signal integrity, through-silicon via array, neural network, particle swarm optimization algorithm

## Abstract

In this research, a signal-integrity control strategy of a through-silicon via (TSV) array for a Chiplet-based system is developed, based on the backpropagation neural network (BP-NN) model and particle swarm optimization algorithm with linear decreasing inertia weight (PSO-LDIW). Based on the HFSS software, the simulation results of the TSV array are obtained. The irregular relationship between design parameters (TSV pitches, height of TSV, radius of TSV, thickness of oxide layer, and offset angle) and signal indexes (return loss, insertion loss, near-end, and far-end crosstalk) is established by the BP-NN model. Then, the design parameters of the TSV array are optimized by the PSO-LDIW algorithm to obtain the desired signal indexes. Based on the optimized design parameters, the effectiveness of the developed signal-integrity control strategy is verified by HFSS simulations. For the three verification cases, the relative errors between the BP-NN-predicted values and the corresponding HFSS simulation values range from 0.31% to 5.02%. The relative deviations of the HFSS results from the desired NEXT, FEXT, and return-loss targets are no greater than 5.72%, while the maximum absolute deviation from the desired insertion-loss target is 0.0160 dB. These results demonstrate the feasibility of the developed strategy for controlling the signal indexes of the TSV array in the tested cases.

## 1. Introduction

With the development of semiconductor technology, the consumer electronics industry has been booming. Due to the high performance and low power, the chips manufactured by the advanced process have dominated a significant portion of the market [1,2]. In the manufacturing process of advanced chips, the three-dimensional integrated circuit (3D IC) is a crucial part [3]. The Chiplet-based system is a kind of complex 3D IC. The through-silicon via (TSV) is adopted to achieve the interconnection in a vertical direction for a Chiplet-based system [4,5], which can improve the performance and energy efficiency of a Chiplet-based system [6,7]. The integrity of the transmitted signal is important for the performance of a Chiplet-based system.

Over the past years, the signal integrity of TSV has been investigated by numerous scholars. The design methodology of the TSV array plays a crucial role in improving the integrity of the transmitted signal [8]. Xu et al. [9] examined the electrical performance of coaxial TSV, a new configuration that provides better signal integrity than other TSV structures. The latency, power, and crosstalk are evaluated and compared between coaxial TSV and the common signal-ground paired TSV. A broadband SPICE model is built to fit the coaxial TSV full-wave solution well, facilitating 3D system design and evaluation. Chandrakar et al. [10] found that adopting feasible bump shapes has a significant impact on the functionality of a Chiplet-based system. To validate the proposed TSV bump structure, the quantitative values of the vias were compared with the finite-element method (FEM) and experimental results, followed by a study of propagation delay, power dissipation, peak noise, insertion, and reflection losses. The proposed via-bump structure showed remarkable consistency with the experimental results. The average deviation is 3.51%. In addition, the conical bump structure can effectively reduce the propagation delay, power dissipation, peak noise, insertion, and reflection losses compared to barrel, cylindrical, and hourglass bumps. Wu et al. [11] presented an accurate magnetic coupling coefficient (k) model for TSV-based 3D transformers. The k factor can be accurately derived from the self-inductance and mutual inductance, which are calculated using various analytical formulas based on the physical geometry. The results of this model agree well with those of the Q3D Extractor and the High-Frequency Structure Simulator (HFSS), with maximum errors of 3.8% and 4.4%, respectively. To meet the requirements for a novel crosstalk reduction scheme for high-density TSV interconnects in a silicon interposer, Yang et al. [12] developed a structure and performance analysis method of TSV with direct ohmic contact between the grounded TSV and the silicon substrate for coupling mitigation. They further extended the structure to a 3 × 3 TSV array and studied its crosstalk performance. The simulation results show that the signal transmission scheme using direct ohmic contact with the grounded TSV can effectively reduce the crosstalk and coupling noise between signal TSVs. Generally, the investigation methods mentioned above are all based on the derived mathematical equations and FEM software to achieve the analyzation and optimization of signal indexes for TSV. However, when the structure of the TSV array becomes more complex, especially when used in the Chiplet-based system, the mathematical derivation will become extremely cumbersome, and the FEM simulation is very time-consuming. Therefore, the high-efficiency signal-integrity control strategy of the TSV array applied in a Chiplet-based system should be investigated.

With the development of artificial intelligence (AI) technology, this has been widely applied to accelerate multidiscipline studies [13]. Chen et al. [14,15,16] developed a strategy based on the particle swarm optimization (PSO) algorithm to control the microstructure of a nickel-based superalloy during hot forging. The strategy consists of three parts, namely, the material model, optimization criteria, and the PSO algorithm. The developed strategy uses the PSO algorithm to solve, does not require a differentiable objective function, and is suitable for various complex situations. The optimal processing parameters were obtained by the developed strategy and verified by hot-forging experiments. The uniform and fine target microstructure can be obtained by the optimized processing parameters, which shows the developed strategy is effective for controlling the microstructural evolution during hot forging of the studied superalloy. Ceyhan et al. [17] introduced a next-generation multi-objective co-optimization approach that jointly leverages machine and human intelligence to solve increasingly demanding challenges at all stages. The approach achieves 10× faster turnaround time and better quality of results compared to a purely human-optimized high-performance CPU implementation at Intel’s 10 nm technology node. Wang et al. [18,19,20] proposed an intelligent optimization method based on the backpropagation neural network (BP-NN) model and PSO algorithm for TSV, aimed to achieve thermal management of the Chiplet-based system. By comparing the errors between simulation values and expected values, it was proved that the developed intelligent design method can achieve thermal management of complex Chiplet-based systems. These investigations cover issues of signal integrity and artificial intelligence optimization in TSV array modeling and simulation. Therefore, the AI model can be used to improve the design efficiency of the TSV array under the constraint of signal integrity.

In this research, a signal-integrity control strategy of a TSV array for a Chiplet-based system is developed based on the BP-NN model and PSO with a linear decreasing inertia weight (PSO-LDIW) algorithm. The innovations can be summarized as follows:

(1) The BP-NN model is established to describe the irregular relationship between design parameters and signal indexes.
(2) The design parameters of the TSV array are optimized by the PSO-LDIW algorithm.
(3) The signal-integrity indexes of the TSV array can be controlled by the developed strategy.

In Section 2, a 3 × 3 TSV array model is established, followed by simulation and analysis results. In Section 3, the signal-integrity control strategy of the TSV array for a Chiplet-based system is presented. In Section 4, the verification and discussion are presented. Finally, the conclusions are given in Section 5.

## 2. Finite-Element Simulation for TSV Array

**Figure 1:**
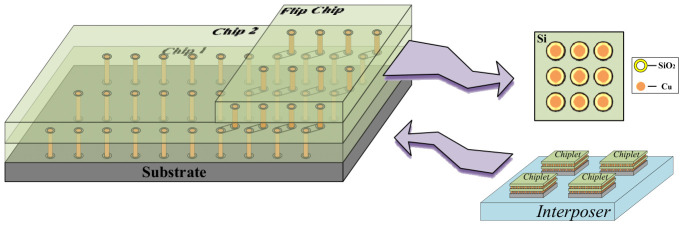
TSV-based 3D Chiplet stacking structure with embedded Cu TSV array.

**Figure 2:**
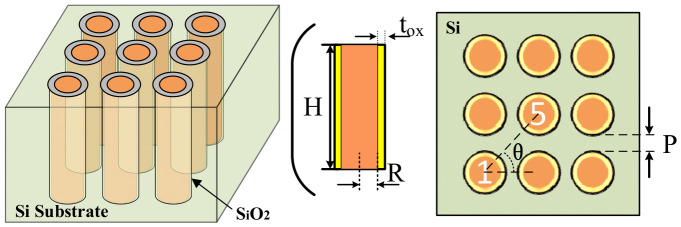
Schematic diagram of the TSV array and its design parameters P, θ, H, R, and t_ox_.

In this research, the HFSS 2024 R2 software is used to obtain the transmitted signal indexes of a TSV array in a Chiplet-based system [21]. Figure 1 illustrates the overall TSV-based interconnect architecture in a Chiplet-based system, which is used as the modeling basis for signal-integrity analysis in this work. As shown in Figure 2, the TSV unit consists of a Cu core, a SiO_2_ insulating layer, and a silicon substrate. According to the design logic, the distance between TSV units (P), the height of the TSV unit (H), the radius of the copper column in the TSV (R), the thickness of the oxide layer of the TSVs (tox), and the offset angle (θ) of the TSV unit are the design parameters, as shown in Figure 2. A finite-element model of a 3 × 3 TSV array is established by HFSS software. The orthogonal experimental method is used to obtain the simulation signal indexes. The ranges of P, H, R, tox and θ are [20, 100], [15, 40], [1.5, 4.5], [0.1, 0.9] μm and [50, 90] deg, respectively. In this research, the signal indexes of TSV1 and TSV5 are simulated according to HFSS software. The signal indexes of the TSV array are return loss (S55(1, 1)), insertion loss (S15(1, 1)), near-end crosstalk (NEXT), and far-end crosstalk (FEXT) [22,23]. The S55(1, 1) corresponds to return loss, while S15(1, 1) corresponds to insertion loss. Lower losses and crosstalk indicate better signal integrity.

**Figure 3:**
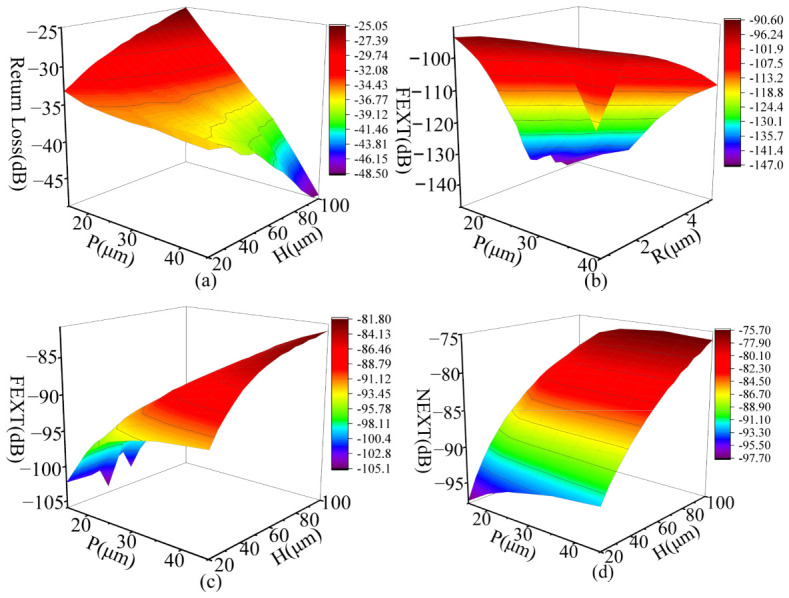
Partial influence of design parameters on signal indexes for the TSV array: (**a**) effect of P and H on return loss; (**b**) effect of P and R on FEXT; (**c**) effect of P and H on FEXT; and (**d**) effect of P and H on NEXT.

According to the design parameters determined by the orthogonal experimental method, the signal indexes can be obtained. The ranges of simulated return loss, insertion loss, NEXT, and FEXT are [−56.587, −17.156], [−0.101, −0.005], [−99.155, −43.837], and [−101.541, −50.864] dB, respectively. Figure 3 shows part of the simulation results. Obviously, the return loss is decreased with increasing P, but it is increased with increasing H, as shown in Figure 3a. According to Figure 3b, the FEXT is decreased with increasing P, but it is firstly increased and then decreased with increasing R. According to Figure 3c, the FEXT is increased with increasing H. The NEXT is increased with increasing P and H, as presented in Figure 3d. Thus, the relationship between the design parameters and signal indexes of the TSV array is irregular and complex.

## 3. Signal Indexes Control Strategy for TSV Array

**Figure 4:**
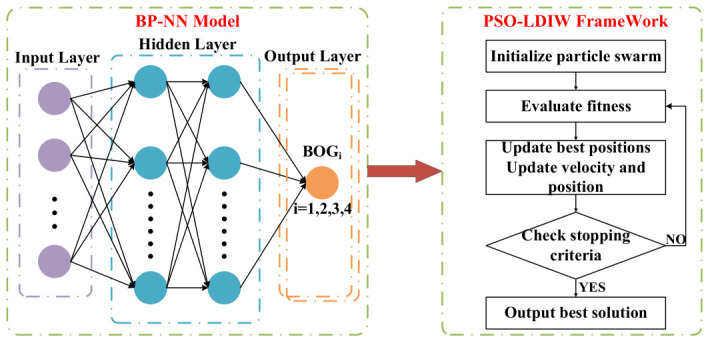
Schematic diagram of control strategy.

In order to efficiently design TSV arrays and meet signal-integrity requirements, the signal-index control strategy of the TSV array for a Chiplet-based system is developed based on the BP-NN model and PSO-LDIW algorithm. The flowchart of the developed strategy is presented in Figure 4. The BP-NN model is established to describe the irregular relationship between the design parameters and signal indexes of the TSV array. The PSO-LDIW algorithm is then adopted to optimize the design parameters according to the desired signal indexes. The multi-objective optimization criterion is formulated by combining the predicted and desired signal indexes. The BP-NN model is used as a computationally efficient surrogate for the HFSS model, which reduces the need for repeated full-wave simulations during optimization. Based on this surrogate model, PSO-LDIW performs a gradient-free inverse search for the TSV design parameters corresponding to the desired signal indexes. Compared with a constant inertia weight, the linearly decreasing inertia weight provides greater exploration capability during the early iterations and gradually strengthens local exploitation during the later iterations.

### 3.1. BP-NN Model Based on Orthogonal Experimental Data

The BP-NN, as a predictive model, has been applied to the modeling and optimization of complex engineering problems. Due to its adaptability, fault tolerance, and robustness, the BP-NN model is chosen to describe the irregular relationship between the design parameters and signal indexes of the TSV array [24,25,26]. In this research, the BP-NN model is established based on the simulation data obtained from the orthogonal experiments. The input layer is composed of the design parameters (P, R, H, t_ox_, and θ), while the outputs are the signal indexes of the TSV array. To evaluate the independent generalization capability of the BP-NN models, the 81 orthogonal experimental samples were randomly divided into three mutually exclusive subsets, and the resulting partition was retained for all four signal indexes. Specifically, 57 samples (70.4%) were used as the training set, 12 samples (14.8%) were used as the validation set, and the remaining 12 samples (14.8%) were reserved as an independent test set. The same sample partition was applied to all four signal indexes. The training set was used to update the network weights, the validation set was used to determine the number of hidden neurons, and the independent test set was used only for the final evaluation of prediction performance. No test sample was involved in network training or model selection. The number of neurons in the hidden layer also affects the prediction accuracy of the BP-NN model. Too few hidden neurons may cause underfitting, whereas too many may lead to overfitting. Therefore, the candidate range of hidden neurons was first determined according to empirical rules, and the final number was selected based on the prediction performance of the validation set.

The requirements of the empirical rule are as follows:

(1) $$n_{x}\geq h\geq n_{y}$$

(2) $$h=\frac{2}{3}\left(n_{x}+n_{y}\right)$$

(3) $$h\leq 2n_{x}$$

where *h* represents the number of neurons in the hidden layer, while *n_x_* and *n_y_*, respectively, denote the number of the input and output layers.

The Mean Absolute Percentage Error (MAPE) used to evaluate the prediction performance is defined as follows:

(4) $$MAPE=\frac{1}{n}\sum_{i=1}^{n}\left|\frac{Y_{i}-{\hat{Y}}_{i}}{Y}\right|\times 100\%$$

where n is the number of samples, $Y_{i}$ represents the actual values obtained from the simulations, and ${\hat{Y}}_{i}$ denotes the corresponding values predicted by the model. Based on the empirical guidelines, candidate BP-NN models with different numbers of hidden neurons were constructed. Each candidate model was trained using the training set, and its prediction performance was evaluated using the validation set. The number of hidden neurons was selected according to the minimum validation error, while the independent test set was not used during this model-selection process. The selected hidden-layer sizes and the corresponding prediction errors on the training, validation, and test sets are summarized in Table 1. The selected numbers of hidden neurons for return loss, insertion loss, NEXT, and FEXT are 7, 8, 7, and 7, respectively.

As shown in Table 1, the validation and independent test errors are of the same order of magnitude, although they are higher than the corresponding training errors, as expected for previously unseen samples. The test samples were not involved in network training or hidden-neuron selection. The test MAPEs are 1.72%, 4.08%, 3.16%, and 4.12% for return loss, insertion loss, NEXT, and FEXT, respectively, and the test MAE of insertion loss is 0.0026 dB. These results indicate that the selected BP-NN models maintain satisfactory predictive capability for previously unseen samples within the investigated parameter ranges.

**Figure 5:**
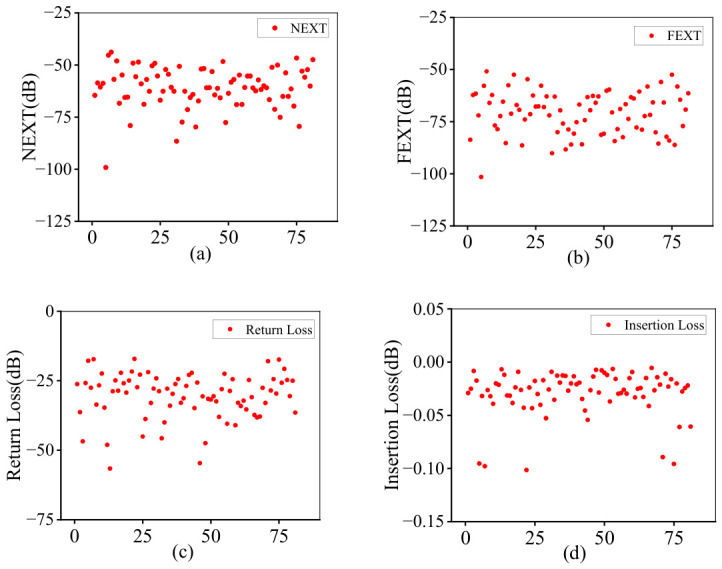
Signal-index data obtained from the 81 orthogonal-design HFSS simulations: (**a**) NEXT; (**b**) FEXT; (**c**) return loss; and (**d**) insertion loss.

The five TSV-array design parameters are the inputs of the BP-NN models, while return loss, insertion loss, NEXT, and FEXT are the corresponding outputs. After the network architectures were selected and their independent prediction performance was evaluated using the hold-out test set, all 81 orthogonal experimental samples were used to retrain the final BP-NN surrogate models for the subsequent PSO-LDIW optimization. The training time of the final BP-NN models was approximately 8 s. The signal indexes obtained from the 81 orthogonal experimental samples are shown in Figure 5. These samples cover the investigated output ranges and provide the simulation database for BP-NN model development.

### 3.2. Formulate Multi-Objective Optimization Criterion

The BP-NN model consists of three parts: an input layer, a hidden layer, and an output layer. Based on the design parameters and desired signal indexes, the BP-NN model can be expressed as

(5) $$h_{1}(k)=W_{1}(k)\cdot \left[Pf(k);Hf(k);Rf(k);\theta f(k);t_{\mathrm{ox}}f(k)\right]$$

(6) $$h_{2}(k)=\frac{1-e^{-h_{1}(k)}}{1+e^{-h_{1}(k)}}$$

(7) $$BOG_{i}f(k)=W_{2}(k)\cdot h_{2}(k),\;i=1,2,3,4$$

where *W*_1_ and *W*_2_ represent weight matrices. *h*_1_ is the input to the hidden layer, while *h*_2_ is the output from the hidden layer. *P*, *H*, *R*, *θ*, and *t_ox_* denote the distance between the TSV unit (*P*), the height of the TSV unit (*H*), the radius of the copper column in the TSV (*R*), the thickness of the oxide layer of the TSVs (*t_ox_*), and the offset angle (*θ*) of the TSV unit. *BOG_i_* (*i* = 1, 2, 3, 4) denote the signal-index values predicted by the BP-NN model, whereas (BOG_i,des) denote the corresponding desired values. *k* denotes the k-th iteration. The multi-objective optimization criterion is represented as

(8) $$fitness=\alpha {\left(BOG_{1}-BOG_{1,\mathrm{des}}\right)}^{2}+\beta {\left(BOG_{2}-BOG_{2,\mathrm{des}}\right)}^{2}+\gamma {\left(BOG_{3}-BOG_{3,\mathrm{des}}\right)}^{2}+\delta {\left(BOG_{4}-BOG_{4,\mathrm{des}}\right)}^{2}$$

where *α*, *β*, *γ*, and *δ* are the weight coefficients of each performance index, which will be determined in the following control strategy section.

### 3.3. Optimal Design Parameters Determined by the PSO-LDIW Algorithm

**Figure 6:**
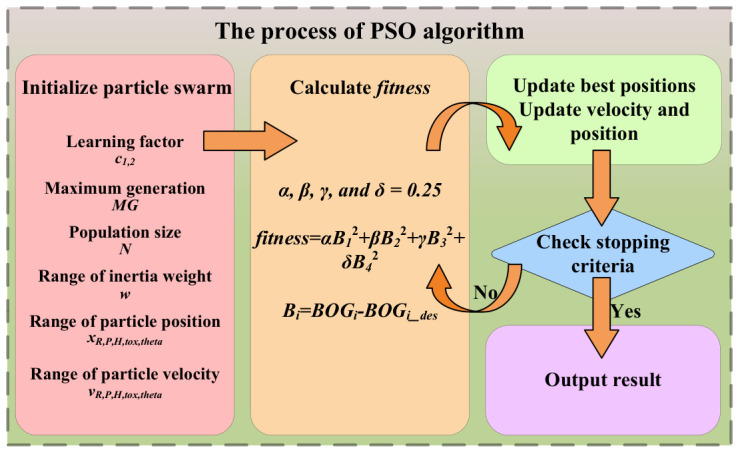
Process of the PSO algorithm.

In this research, the PSO-LDIW algorithm is used to realize the signal indexes control of the TSV array for a Chiplet-based system. The flowchart of the PSO-LDIW algorithm, based on the established BP-NN model, is presented in Figure 6. Firstly, the parameters of the PSO-LDIW algorithm are initialized, as shown in TABLE II. Then, the objective function of each particle is evaluated, and the individual best position of each particle, as well as the global best position of the entire swarm, are determined. Finally, the velocity and position of each particle are updated, and whether or not the target condition is achieved is subsequently evaluated.

Generally, each signal-integrity index should be close to its desired value. The desired values in Cases A–C were selected within the ranges of the magnitudes of the signal indexes covered by the 81 HFSS samples, so that the optimization remained within the surrogate-model domain. The three cases represent different levels of design stringency. Case A imposes the most stringent crosstalk-isolation and insertion-loss targets, Case B represents an intermediate condition, and Case C adopts relatively relaxed crosstalk targets, together with a higher allowable insertion loss. The return-loss targets of 18–20 dB provide two matching-performance levels. Thus, the three cases evaluate the proposed method under different combinations of signal-integrity requirements, rather than under a single target condition.

The accuracy of the optimized signal indexes is influenced by the weight coefficients α, β, γ, and δ. Therefore, different combinations of weight coefficients were evaluated according to the deviations between the BP-NN-predicted signal indexes and their corresponding desired values.

For consistency with the desired values listed in Table 2, the magnitudes of the dB-valued signal indexes are used in the following calculations. For NEXT, FEXT, and return loss, the relative target deviation is defined as

(9) $$RE_{\mathrm{opt},i}=\frac{\left|Y_{\mathrm{pred},i}-Y_{\mathrm{des},i}\right|}{\left|Y_{\mathrm{des},i}\right|}\times 100\%$$

where ($Y_{\mathrm{pred},i}$) and ($Y_{\mathrm{des},i}$) denote the BP-NN-predicted value and the desired value of the (i)-th signal index, respectively. Because the desired insertion-loss values are close to zero, a small absolute difference may produce a disproportionately large percentage deviation. Therefore, the absolute target deviation is used for insertion loss, and is defined as

(10) $$AE_{\mathrm{opt},\mathrm{IL}}=\left|Y_{\mathrm{pred},\mathrm{IL}}-Y_{\mathrm{des},\mathrm{IL}}\right|$$

The target deviations obtained using different combinations of weight coefficients are summarized in Table 3. During the k-th generation, the inertia weight decreases linearly from 0.9 to 0.4 over the 300 generations.

(11) $$w\left(k\right)=w_{max}-\frac{w_{max}-w_{min}}{MG}k$$

Based on the comparison in Table 3, the weight coefficients (α), (β), (γ), and (δ) were selected as 0.3, 0.2, 0.4, and 0.1, respectively. With these coefficients, the relative target deviations of the BP-NN-predicted NEXT, FEXT, and return loss from their desired values are 2.51%, 1.48%, and 5.75%, respectively, while the absolute target deviation of insertion loss is 0.0143 dB.

**Figure 7:**
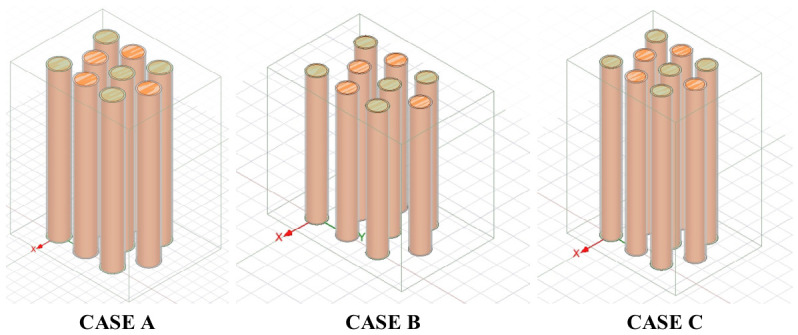
Model diagrams of CASE (**A**), CASE (**B**) and CASE (**C**).

**Figure 8:**
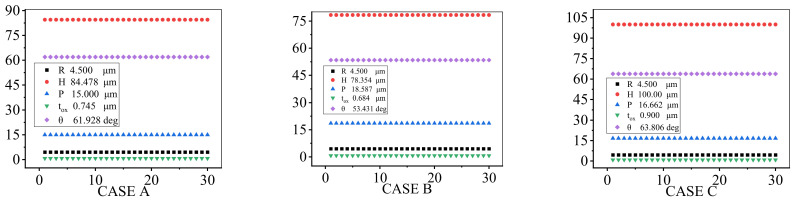
Thirty sets of independent repeated validation data with the design parameters.

Figure 7 shows the complete case model under three sets of design parameters. In order to prove the stability of the developed strategy, the developed strategy is independently run 30 times. The running results with design parameters are shown in Figure 8. Obviously, the variation range of the 30 independent running results is very small, indicating that the developed strategy is stable.

## 4. Verification and Discussion

### 4.1. Verification

The optimized TSV structures for Cases A–C were reconstructed and simulated in HFSS. The BP-NN-predicted values obtained during the PSO optimization were then compared with the corresponding HFSS simulation values, as summarized in Table 4. The three optimized verification cases were not included in the 81-sample orthogonal dataset, and therefore provide an external HFSS verification of the final surrogate-assisted optimization procedure.

Three quantities are involved in the verification: the desired value, the value predicted by the BP-NN model during PSO optimization, and the value obtained by simulating the optimized structure in HFSS. For consistency with the optimization targets, the magnitudes of the dB-valued signal indexes are used in the error calculations.

The relative error between the BP-NN-predicted value and the corresponding HFSS simulation value is defined as

(12) $$RE_{\mathrm{model},i}=\frac{\left|Y_{\mathrm{pred},i}-Y_{\mathrm{HFSS},i}\right|}{\left|Y_{\mathrm{HFSS},i}\right|}\times 100\%$$

where (Y_pred,i) and (Y_HFSS,i) represent the BP-NN-predicted value and the HFSS simulation value of the (i)-th signal index, respectively. This error evaluates the predictive accuracy of the BP-NN surrogate model.

The relative deviation of the HFSS simulation value from the desired target is defined as

(13) $$RE_{\mathrm{target},i}=\frac{\left|Y_{\mathrm{HFSS},i}-Y_{\mathrm{des},i}\right|}{\left|Y_{\mathrm{des},i}\right|}\times 100\%$$

where (Y_des,i) denotes the desired value of the (i)-th signal index. This deviation evaluates the target-control performance of the proposed strategy.

Because the desired insertion-loss values are close to zero, the corresponding percentage deviations may be amplified. Therefore, the absolute target deviation of insertion loss is additionally defined as

(14) $$AE_{\mathrm{target},\mathrm{IL}}=\left|Y_{\mathrm{HFSS},\mathrm{IL}}-Y_{\mathrm{des},\mathrm{IL}}\right|$$

As shown in Table 5, among the 12 comparisons between the BP-NN predictions and the corresponding HFSS simulation values, 11 relative errors are below 5%, while the FEXT prediction error in Case A is 5.02%. Therefore, the BP-NN prediction errors range from 0.31% to 5.02%.

**Figure 9:**
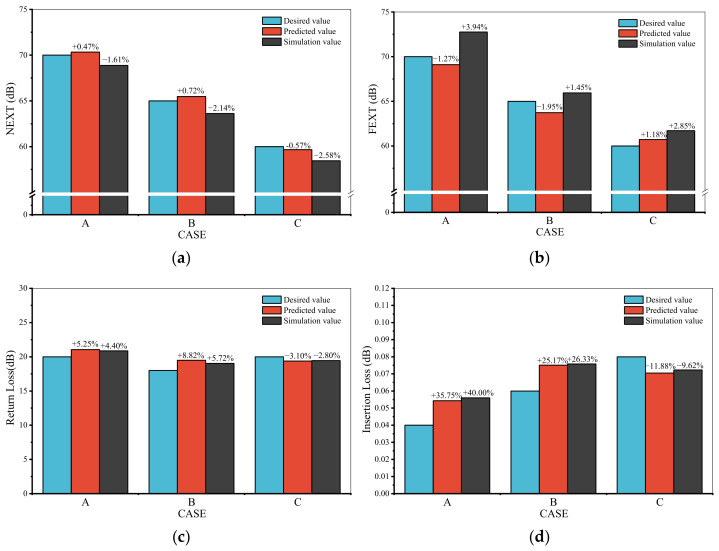
Comparison of the desired, BP-NN-predicted, and HFSS-simulated signal indexes: (**a**) NEXT; (**b**) FEXT; (**c**) return loss; and (**d**) insertion loss. The signs of the percentage annotations indicate whether the predicted or simulated values are above or below the corresponding desired values.

Regarding the target-control performance, all nine relative deviations associated with NEXT, FEXT, and return loss are below 8%, with a maximum value of 5.72%. In contrast, the relative deviations of insertion loss range from 9.63% to 40.00%, which are considerably larger than those of the other three signal indexes. Because the desired insertion-loss values are close to zero, the percentage deviations are sensitive to small absolute variations. The corresponding absolute deviations are 0.0077–0.0160 dB. Therefore, both relative and absolute deviations are reported for insertion loss. Figure 9 further compares the desired, BP-NN-predicted, and HFSS-simulated values.

Figure 9 compares the desired, BP-NN-predicted, and HFSS-simulated signal indexes. For the HFSS simulation results, the relative deviations from the desired values are 1.61–2.58% for NEXT, 1.45–3.94% for FEXT, and 2.80–5.72% for return loss. The relative deviations of insertion loss are larger because its desired values are close to zero. Nevertheless, the corresponding absolute deviations are only 0.0077–0.0160 dB. Therefore, both relative percentage deviation and absolute deviation are considered when evaluating the target-control performance of the proposed strategy.

### 4.2. Discussion

This article proposes a control strategy for the design parameters of TSV arrays in 3D-IC, in order to obtain excellent signal integrity. HFSS software is used to obtain finite-element simulation data, and orthogonal experimental design is used to screen the data for training the BP-NN model. The BP-NN model is trained to describe the complex relationship between the design parameters and performance indexes of the TSV array model. Based on the importance of performance indexes, optimization criteria are established. Finally, the PSO algorithm is used to optimize the design parameters to improve signal integrity.

For the three verification cases, the maximum relative error between the BP-NN-predicted values and the corresponding HFSS simulation values is 5.02%. This comparison evaluates the predictive accuracy of the BP-NN surrogate model.

For target-control performance, the maximum relative deviations of the HFSS results from the desired NEXT, FEXT, and return-loss values are 2.58%, 3.94%, and 5.72%, respectively. The relative deviations of insertion loss range from 9.63% to 40.00% because its desired values are close to zero, whereas the corresponding absolute deviations range from 0.0077 to 0.0160 dB. These results demonstrate satisfactory predictive and target-control performance for the three verification cases, while also indicating that both relative and absolute deviations should be considered for near-zero insertion-loss values.

Among the reported results, insertion loss represents the least favorable target-control metric. Its relative target deviations range from 9.63% to 40.00%, with the largest value occurring in Case A. These relatively large percentage deviations are retained in the analysis, rather than excluded; however, they correspond to absolute deviations of only 0.0077–0.0160 dB because the desired insertion-loss values are close to zero.

The validated applicability of the current BP-NN model is limited to the 3 × 3 Cu TSV-array topology, the SiO_2_ insulation and silicon-substrate configuration, and to the ranges of P, H, R, t_ox_, and θ specified in Section 2 under the same HFSS material, excitation, and boundary settings. The trained BP-NN model should not be directly applied to different array sizes, TSV arrangements, material systems, or parameter ranges. For another TSV-array configuration, the proposed BP-NN–PSO-LDIW workflow remains applicable, but a new HFSS dataset and model retraining are required.

Although physical experimental measurements are not included in the present study, the proposed strategy is verified through a simulation-based procedure. The original dataset used for BP-NN model development is generated by full-wave finite-element simulations in HFSS, and the trained surrogate models are further evaluated using an independent test set. In addition, after the design parameters are obtained by PSO-LDIW, the optimized TSV structures for Cases A–C are reconstructed and simulated again in HFSS. These verification cases are not included in the original 81-sample orthogonal dataset, and therefore provide an external simulation-based verification of the surrogate-assisted optimization procedure.

It should be noted that the present work focuses on developing an efficient inverse-design and signal-integrity control framework for TSV arrays. The HFSS-based verification demonstrates the feasibility of the proposed method under the investigated TSV topology, material configuration, parameter ranges, and electromagnetic simulation settings. Nevertheless, fabrication and measurement of TSV-array samples would provide further experimental validation of the proposed strategy. This will be considered in future work.

To evaluate the contribution of the PSO-LDIW algorithm, it was compared with standard PSO, random search, and a genetic algorithm under the same optimization conditions. The genetic algorithm used a population size of 50, 300 generations, a crossover probability of 0.8, and the same design-parameter bounds, fitness function, and evaluation budget as the PSO-LDIW algorithms. The standard PSO used a constant inertia weight of 0.65, while the other PSO parameters were identical to those of PSO-LDIW. For random search, 15,000 candidate designs were uniformly sampled within the same parameter bounds, equal to the number of surrogate-model evaluations used in each PSO run. Each method was independently executed 30 times for Cases A–C, and the mean final fitness value and mean computation time were calculated.

As shown in Table 6, PSO-LDIW achieves the lowest mean final fitness values for all three cases. Compared with standard PSO, the mean final fitness values are reduced by approximately 11.9%, 4.2%, and 10.4% for Cases A, B, and C, respectively. Compared with random search, the corresponding reductions are approximately 16.6%, 14.8%, and 16.2%. Compared with the genetic algorithm, the reductions are approximately 34.8%, 26.8%, and 39.9%. The computational times of the four methods remain of the same order of magnitude. These results support the selection of PSO-LDIW within the proposed BP-NN-assisted inverse-design framework.

In addition, on an Intel(R) Xeon(R) Gold 6226R CPU operating at 2.9 GHz, one BP-NN surrogate evaluation requires less than 2 s, whereas one HFSS simulation requires approximately 29 s. One complete PSO-LDIW optimization run requires approximately 802.96 s, and 30 independent runs require approximately 24,088.8 s. The surrogate-assisted framework therefore avoids repeatedly invoking the full-wave HFSS model during the iterative search. Compared with conventional manual trial-and-error design, the proposed method automatically determines the structural parameters according to the desired performance indexes.

## 5. Conclusions

This article proposes a control strategy for TSV arrays based on the BP-NN model and PSO-LDIW algorithm for optimizing in 3D-IC. This method improves signal integrity by optimizing the design parameters of the TSV array. The main conclusions of the study are as follows:

(1) A detailed finite-element model of TSV array is established. The relationship between design parameters and performance indexes is analyzed, revealing irregular and complex characteristics.
(2) For the three verification cases, the maximum relative error between the BP-NN predictions and the corresponding HFSS simulation results is 5.02%. The maximum relative deviation of the HFSS results from the desired NEXT, FEXT, and return-loss targets is 5.72%, while the maximum absolute deviation from the desired insertion-loss target is 0.0160 dB. These results verify the predictive accuracy and target-control capability of the proposed method for the tested cases. The current trained surrogate model is applicable to the investigated TSV topology and parameter ranges; other TSV configurations require new simulation data and model retraining.
(3) The proposed design method reduces dependence on expert experience. One complete PSO-LDIW optimization run requires approximately 802.96 s (less than 14 min), while each surrogate-model evaluation is substantially faster than an HFSS simulation.

Future work will further consider the fabrication and measurement of TSV-array samples to experimentally validate the proposed signal-integrity control strategy.

## Figures and Tables

**Table 1:** Prediction performance of the selected BP-NN models on the training, validation, and independent test sets.

Signal Index	Hidden Neurons	Training MAPE (%)	Validation MAPE (%)	Test MAPE (%)	Insertion-Loss Test MAE (dB)
Return loss	7	0.42	1.36	1.72	-
Insertion loss	8	1.18	3.41	4.08	0.0026
NEXT	7	1.43	2.79	3.16	-
FEXT	7	1.76	3.55	4.12	-

**Table 2:** Parameter settings for PSO algorithm.

Parameter		Setting
Learning factor		*c*_1_ = *c*_2_ = 2
Maximum generation		*MG* = 300
Population size		*N* = 50
Range of inertia weight		*w* ∈ [0.4, 0.9]
Range of particle position		$\begin{array}{l} x_{R}\in [1.5,4.5],\;x_{P}\in [20,100],\;x_{H}\in [15,40],\\ x_{t_{\mathrm{ox}}}\in [0.1,0.9],\;x_{\theta }\in [50,90] \end{array}$
Range of particle velocity		$\begin{array}{l} v_{R}\in [-0.01,0.01],\;v_{P}\in [-0.25,0.25],\;v_{H}\in [-0.05,0.05],\\ v_{t_{\mathrm{ox}}}\in [-0.05,0.05],\;v_{\theta }\in [-0.05,0.05] \end{array}$
Desired Value	Case A	*BOG*_1_*__des_* = 20, *BOG*_2_*__des_* = 0.04, *BOG*_3_*__des_* = 70, *BOG*_4_*__des_* = 70
	Case B	*BOG*_1_*__des_* = 18, *BOG*_2_*__des_* = 0.06, *BOG*_3_*__des_* = 65, *BOG*_4_*__des_* = 65
	Case C	*BOG*_1_*__des_* = 20, *BOG*_2_*__des_* = 0.08, *BOG*_3_*__des_* = 60, *BOG*_4_*__des_* = 60

**Table 3:** Target deviations under different weight coefficients.

Case	Weight Coefficient				Relative Target Deviation (%)			Absolute Target Deviation (dB)
	*α*	*β*	*γ*	*δ*	NEXT	FEXT	Return Loss	Insertion Loss
1	0.25	0.25	0.25	0.25	2.16%	0.81%	6.82%	0.0146
2	0.25	0.1	0.4	0.25	2.56%	2.26%	4.86%	0.0098
3	0.4	0.1	0.3	0.2	1.51%	2.27%	6.61%	0.0073
4	0.3	0.2	0.25	0.25	1.84%	1.10%	7.17%	0.0101
5	0.3	0.2	0.3	0.2	2.10%	1.24%	6.64%	0.0151
6	0.3	0.2	0.4	0.1	2.51%	1.48%	5.75%	0.0143

**Table 4:** Desired, BP-NN-predicted, and HFSS-simulated signal indexes for the three verification cases.

		NEXT (dB)	FEXT (dB)	Return Loss (dB)	Insertion Loss (dB)
CASE A	Desired value	70	70	20	0.040
	BP-NN Predicted value	70.33	69.11	21.05	0.0543
	HFSS Simulation value	68.87	72.76	20.88	0.0560
CASE B	Desired value	65	65	18	0.060
	BP-NN Predicted value	65.47	63.73	19.49	0.0751
	HFSS Simulation value	63.61	65.94	19.03	0.0758
CASE C	Desired value	60	60	20	0.080
	BP-NN Predicted value	59.66	60.71	19.38	0.0705
	HFSS Simulation value	58.45	61.71	19.44	0.0723

Note: The magnitudes of the signal indexes in dB are listed for consistency with the optimization targets.

**Table 5:** Prediction errors of the BP-NN model and deviations of the HFSS results from the desired targets.

Case	Signal Index	BP-NN Prediction Error RE_Model (%)	HFSS Target Deviation RE_Target (%)	HFSS Target Absolute Deviation (dB)
A	NEXT	2.12	1.61	1.1300
A	FEXT	5.02	3.94	2.7600
A	Return Loss	0.81	4.40	0.8800
A	Insertion Loss	3.04	40.00	0.0160
B	NEXT	2.92	2.14	1.3900
B	FEXT	3.35	1.45	0.9400
B	Return Loss	2.42	5.72	1.0300
B	Insertion Loss	0.92	26.33	0.0158
C	NEXT	2.07	2.58	1.5500
C	FEXT	1.62	2.85	1.7100
C	Return Loss	0.31	2.80	0.5600
C	Insertion Loss	2.49	9.63	0.0077

Note: For completeness, the absolute target deviations of all four signal indexes are also listed in Table 5, while the absolute deviation is used as the primary supplementary metric only for insertion loss.

**Table 6:** Comparison of the optimization performance of PSO-LDIW, standard PSO, the genetic algorithm, and random search over 30 independent runs.

Method ^1^	Case A Final Fitness	Case B final Fitness	Case C Final Fitness	Average Time per Run (s)
PSO-LDIW	0.457	0.941	0.223	802.96
Standard PSO	0.519	0.982	0.249	789.40
Random search	0.548	1.104	0.266	844.70
Genetic algorithm	0.701	1.286	0.371	615.30

^1^ All methods use the same BP-NN surrogate model, design-parameter bounds, fitness function, and a budget of 15,000 surrogate-model evaluations per run. Standard PSO uses a constant inertia weight of 0.65. The genetic algorithm used a population size of 50, 300 generations, a crossover probability of 0.8, and the same design-parameter bounds, fitness function, and evaluation budget as the PSO-LDIW algorithms.

## Data Availability

No new data were created or analyzed in this study.

## References

[1] Tsai Y.-C., Lee C.-H., Chang H.-C., Liu J.-H., Hu H.-W., Ito H., Kim Y.S., Ohba T., Chen K.-N. (2021). Electrical Characteristics and Reliability of Wafer-on-Wafer (WOW) Bumpless Through-Silicon Via. IEEE Trans. Electron Devices.

[2] Peng X., Kaul A., Bakir M.S., Yu S. (2021). Heterogeneous 3-D Integration of Multitier Compute-in-Memory Accelerators: An Electrical-Thermal Co-Design. IEEE Trans. Electron Devices.

[3] Banerjee K., Souri S.J., Kapur P., Saraswat K.C. (2001). 3-D ICs: A Novel Chip Design for Improving Deep-Submicrometer Interconnect Performance and Systems-on-Chip Integration. Proc. IEEE.

[4] Song T., Liu C., Peng Y., Lim S.K. (2016). Full-Chip Signal Integrity Analysis and Optimization of 3-D ICs. IEEE Trans. Very Large Scale Integr. (VLSI) Syst..

[5] Van der Plas G., Limaye P., Mercha A., Oprins H., Torregiani C., Thijs S., Linten D., Stucchi M., Guruprasad K., Velenis D. Design Issues and Considerations for Low-Cost 3D TSV IC Technology. Proceedings of the 2010 IEEE International Solid-State Circuits Conference—(ISSCC).

[6] Rahman A., Reif R. (2000). System-Level Performance Evaluation of Three-Dimensional Integrated Circuits. IEEE Trans. Very Large Scale Integr. (VLSI) Syst..

[7] Suzano J., Abouzeid F., Di Natale G., Philippe A., Roche P. (2024). On Hardware Security and Trust for Chiplet-Based 2.5D and 3D ICs: Challenges and Innovations. IEEE Access.

[8] Kim H., Park J., Lee S., Kim J., Ahn S. (2023). Signal Integrity Analysis of Through-Silicon-Via (TSV) with Passive Equalizer to Separate Return Path and Mitigate the Inter-Symbol Interference (ISI) for Next Generation High Bandwidth Memory. IEEE Trans. Compon. Packag. Manuf. Technol..

[9] Xu Z., Lu J.-Q. (2012). Three-Dimensional Coaxial Through-Silicon-Via (TSV) Design. IEEE Electron Device Lett..

[10] Chandrakar S., Gupta D., Majumder M.K., Kaushik B.K. (2022). Performance Analysis of Bump in Tapered TSV: Impact on Crosstalk and Power Loss. IEEE Open J. Nanotechnol..

[11] Wu H., Dong G., Xiong W., Zhi C., Li S., Zhu Z., Yang Y. (2022). Accurate Magnetic Coupling Coefficient Modeling of 3-D Transformer Based on TSV. IEEE Microw. Wirel. Compon. Lett..

[12] Yang D.C., Li E.P., Jun L., Wei X.C., Xie J.Y., Swaminathan M. Crosstalk Reduction in TSV Arrays with Direct Ohmic Contact between Metal and Silicon-Substrate. Proceedings of the 2014 International Symposium on Electromagnetic Compatibility.

[13] Kim K., Park H., Lho D., Kim M., Son K., Son K., Kim S., Shin T., Choi S., Kim J. Deep Reinforcement Learning-Based Through Silicon Via (TSV) Array Design Optimization Method Considering Crosstalk. Proceedings of the 2020 IEEE Electrical Design of Advanced Packaging and Systems (EDAPS).

[14] Chen D., Wang X., Yang Y., Li D., Li G. (2023). Intelligent Codesign Strategy for Thermal Management and Cost Control of 3-D Integrated System with TTSV. IEEE Trans. Electron Devices.

[15] Cui Y., Wang X., Chen D., Li D., Yang Y. (2025). Intelligent Optimization Design of GS-TSV with RDLs for Chiplet-Based Integrated System in IoT. IEEE Internet Things J..

[16] Jiang R., Wang X., Chen D., Li D., Yang Y. (2025). A High-Efficiency Crosstalk Control Method of the Staggered High-Speed via Array in PCB for Chiplet-Based System. IEEE Trans. Electromagn. Compat..

[17] Ceyhan A., Quijas J., Jain S., Liu H.-Y., Gifford W.E., Chakravarty S. Machine Learning-Enhanced Multi-Dimensional Co-Optimization of Sub-10nm Technology Node Options. Proceedings of the 2019 IEEE International Electron Devices Meeting (IEDM).

[18] Wang X., Yang Y., Chen D., Li D. (2023). Intelligent Design Method of Thermal Through Silicon via for Thermal Management of Chiplet-Based System. IEEE Trans. Electron Devices.

[19] Wang X., Huang J., Chen D., Li D., Yang Y. (2024). Electro-Thermal-Stress Multiphysical Field Coupling Optimization Design for Coaxial Through Silicon Via Array. IEEE Trans. Compon. Packag. Manuf. Technol..

[20] Wang X., Yang Y., Chen D., Li D. (2023). A High-Efficiency Design Method of TSV Array for Thermal Management of 3-D Integrated System. IEEE Trans. Comput.-Aided Des. Integr. Circuits Syst..

[21] Fang X., Yu Y., Xu K., Peng X. (2018). TSV-Defect Modeling, Detection and Diagnosis Based on 3-D Full Wave Simulation and Parametric Measurement. IEEE Access.

[22] Dahl D., Reuschel T., Preibisch J.B., Duan X., Ndip I., Lang K.-D., Schuster C. (2016). Efficient Total Crosstalk Analysis of Large via Arrays in Silicon Interposers. IEEE Trans. Compon. Packag. Manuf. Technol..

[23] Chandrakar S., Solanki K., Gupta D., Majumder M.K. (2024). Electrical Modeling and Performance Analysis of Cu and CNT Based TSV-Bump-RDL. IEEE Trans. Nanotechnol..

[24] Zhang Y., Gao X., Katayama S. (2015). Weld Appearance Prediction with BP Neural Network Improved by Genetic Algorithm during Disk Laser Welding. J. Manuf. Syst..

[25] Xiang Q., Zhang X., Sun Y. Calculation of BSRM’s Inductance with PSO-BPNN. Proceedings of the 30th Chinese Control Conference.

[26] Yang T., Qin H., Ning B., Xu Y., Yao Y., You Z. Application of T-SNE and PSO-BPNN for Identification of Penetration State in Ship GMAW. Proceedings of the 2023 IEEE 6th International Conference on Pattern Recognition and Artificial Intelligence (PRAI).

